# Supplementary material for: EVL and MIM/MTSS1 regulate actin cytoskeletal remodeling to promote dendritic filopodia in neurons
Source: J Cell Biol. 2023 Feb 24;222(5):e202106081. doi: 10.1083/jcb.202106081 (PMC9998662; doi:10.1083/jcb.202106081)
Supplement: Table S1 — shows plasmids generated and used in this study. [file JCB_202106081_TableS1.docx]

**TABLE 1: Plasmids generated and used**

| PLASMID NAME | SOURCE | IDENTIFIER |
| --- | --- | --- |
| pCAAGs PSD95-FingR GFP | this paper |  |
| pCAAGs PSD95-FingR mRuby2 | this paper |  |
| pCIB mRuby2-Arp3 | this paper |  |
| pCIB MRLC-mRuby2 | (Padilla-Rodriguez et al., 2018) | RRID:Addgene_84383 |
| pCW57.1 mCherry-FP4-MITO (all-in-one dox-inducible) | this paper |  |
| pCW57.1 mCherry-AP4-MITO (all-in-one dox-inducible) | this paper |  |
| pCIB EGFP-LifeAct | (Padilla-Rodriguez et al., 2018) | RRID:Addgene_84383 |
| pLKO.1 TurboRFP cloning vector | this paper |  |
| pLKO.1 n.t. TurboRFP (anti-sense CGAGGGCGACTTAACCTTAGG) | this paper |  |
| pLKO.1 sh*Enah* #1 TurboRFP (anti-sense TTAAAGGGCGATTGTCTTCTG) | this paper, seq. source: TRCN0000091680 |  |
| pLKO.1 sh*Enah* #2 TurboRFP (anti-sense TATGCAGTGTTCGACTTGCTC) | this paper, seq. source: TRCN0000091682 |  |
| pLKO.1 sh*Evl* #1 TurboRFP (anti-sense TTGTTCATTTCTTCCATGAGG) | this paper, seq. source: TRCN0000091075 |  |
| pLKO.1 sh*Evl* #2 TurboRFP (anti-sense TTGGAAGCCATCTAATAAAGC) | this paper, seq. source: TRCN0000091073 |  |
| pLKO.1 sh*Vasp* TurboRFP (anti-sense TTCTCACAGGTTCACTCTGGG) | this paper, seq. source: TRCN0000054420 |  |
| pLKO.1 sh*Mtss1* TurboRFP (anti-sense CCCACGACTCAGGATTCATAT) | this paper, seq. source: TRCN0000279395 |  |
| pCIB EGFP | this paper |  |
| pCIB EGFP-MENA | this paper |  |
| pCIB EGFP-EVL | this paper |  |
| pCIB mRuby2-LifeAct | (Parker et al., 2018) | RRID:Addgene_84384 |
| pEIB mEmerald | this paper |  |
| pEIB mEmerald-EVL | this paper |  |
| pEIB mEmerald-ΔEVH1-EVL | this paper |  |
| pEIB mEmerald-EVH1 (EVL) alone | this paper |  |
| pEIB mEmerald-ΔPFN-EVL | this paper |  |
| pEIB mEmerald-PRR (EVL) alone | this paper |  |
| pEIB mEmerald-ΔGAB-EVL | this paper |  |
| pEIB mEmerald-ΔPFNΔGAB-EVL (ΔActinPoly) | this paper |  |
| pEIB mEmerald-ΔGABΔFAB-EVL | this paper |  |
| pEIB mEmerald-EVH2 (EVL) alone | this paper |  |
| pLL7.0 Venus-iLID-CAAX (from KRas4B) | (Guntas et al., 2015) | RRID:Addgene_60411 |
| pLL7.0 Venus-iLID-Mito (from ActA) | (Guntas et al., 2015) | RRID:Addgene_60413 |
| pCIB tgRFPt-SspB(R73Q)-EVL | this paper |  |
| pCIB tgRFPt-SspB(R73Q)-ΔEVH1-EVL | this paper |  |
| pCIB tgRFPt-SspB(R73Q)-ΔPFN-EVL | this paper |  |
| pCIB tgRFPt-SspB(R73Q)-ΔPFNΔGAB-EVL (ΔActinPoly) | this paper |  |
| pCIB iRFP670-LifeAct | (Padilla-Rodriguez et al., 2018) | RRID:Addgene_84385 |
| pCMV6 MIM-myc-FLAG | Origene | MR210506 |
| pCMV 3xFLAG | (Parker et al., 2013) |  |
| pEFS GFP-EVL | this paper |  |
| pEFS GFP-ΔEVH1 | this paper |  |
| pEFS GFP-ΔPRR | this paper |  |
| pCAGGs GFP-ΔIBAR-MIM LPPSP | (Galbraith et al., 2018) |  |
| pCAGGs GFP-ΔIBAR-MIM AGGGG | this paper |  |
| pEF1 EVH1-mychis6 | this paper |  |
| pEF1-myc-his6 B | Invitrogen, V92120 |  |
| pLV MIM-mRuby2 | this paper |  |
| pLV MIM-iRFP670 | this paper |  |
| pLKO sh*Mtss1* TurboRFP | this paper |  |
| pLV MIM-tgRFPt-SspB(R73Q) | this paper |  |
| pCIB mEmerald-Arp3 | this paper |  |
| pCIB iRFP670-EVL | this paper |  |
| pMD2.G | Didier Trono | RRID:Addgene_12259 |
| psPAX2 | Didier Trono | RRID:Addgene_12260 |
