# Supplementary figures and images for: EVL and MIM/MTSS1 regulate actin cytoskeletal remodeling to promote dendritic filopodia in neurons
Source: J Cell Biol. 2023 Feb 24;222(5):e202106081. doi: 10.1083/jcb.202106081 (PMC9998662; doi:10.1083/jcb.202106081)

Fig 1F: MENA/VASP/EVL expression in cultured neurons

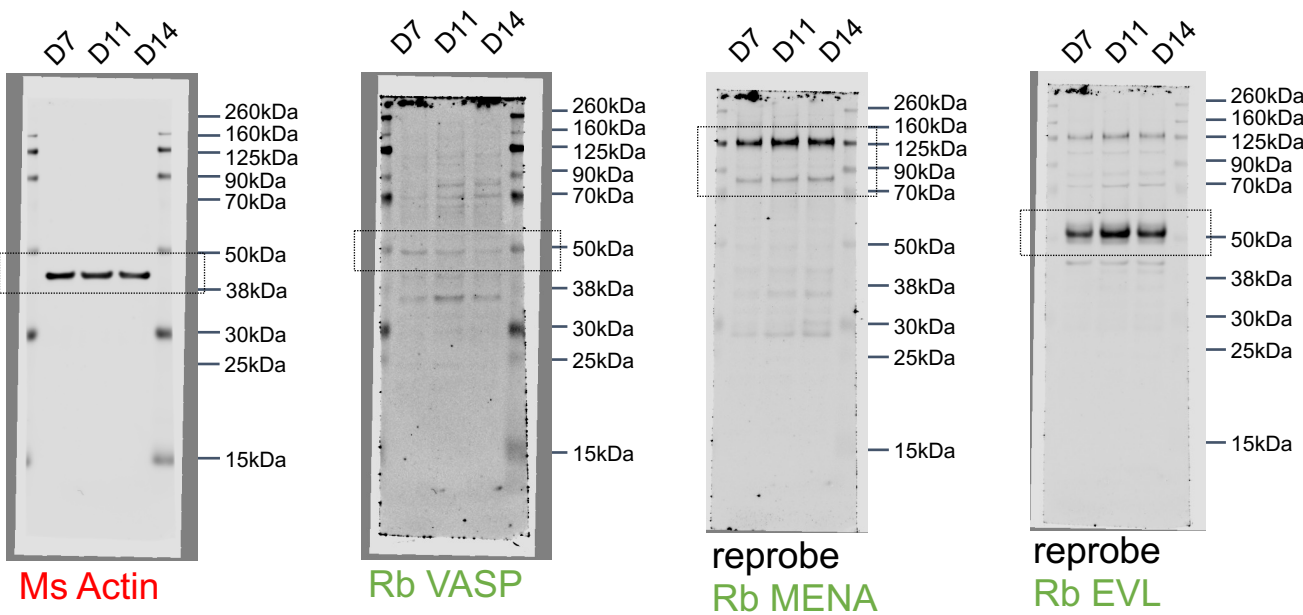

Supplement: SourceData F1 — is the source file for Fig. 1. [file JCB_202106081_SourceDataF1.pdf]

Fig 5B: Expression of EVL constructs

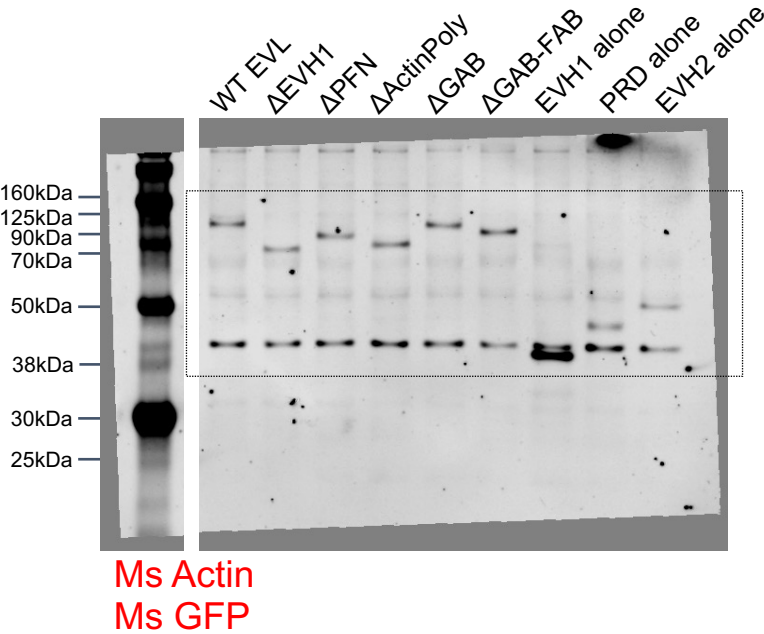

Supplement: SourceData F5 — is the source file for Fig. 5. [file JCB_202106081_SourceDataF5.pdf]

Fig S2G-I: MENA/VASP/EVL shRNAs

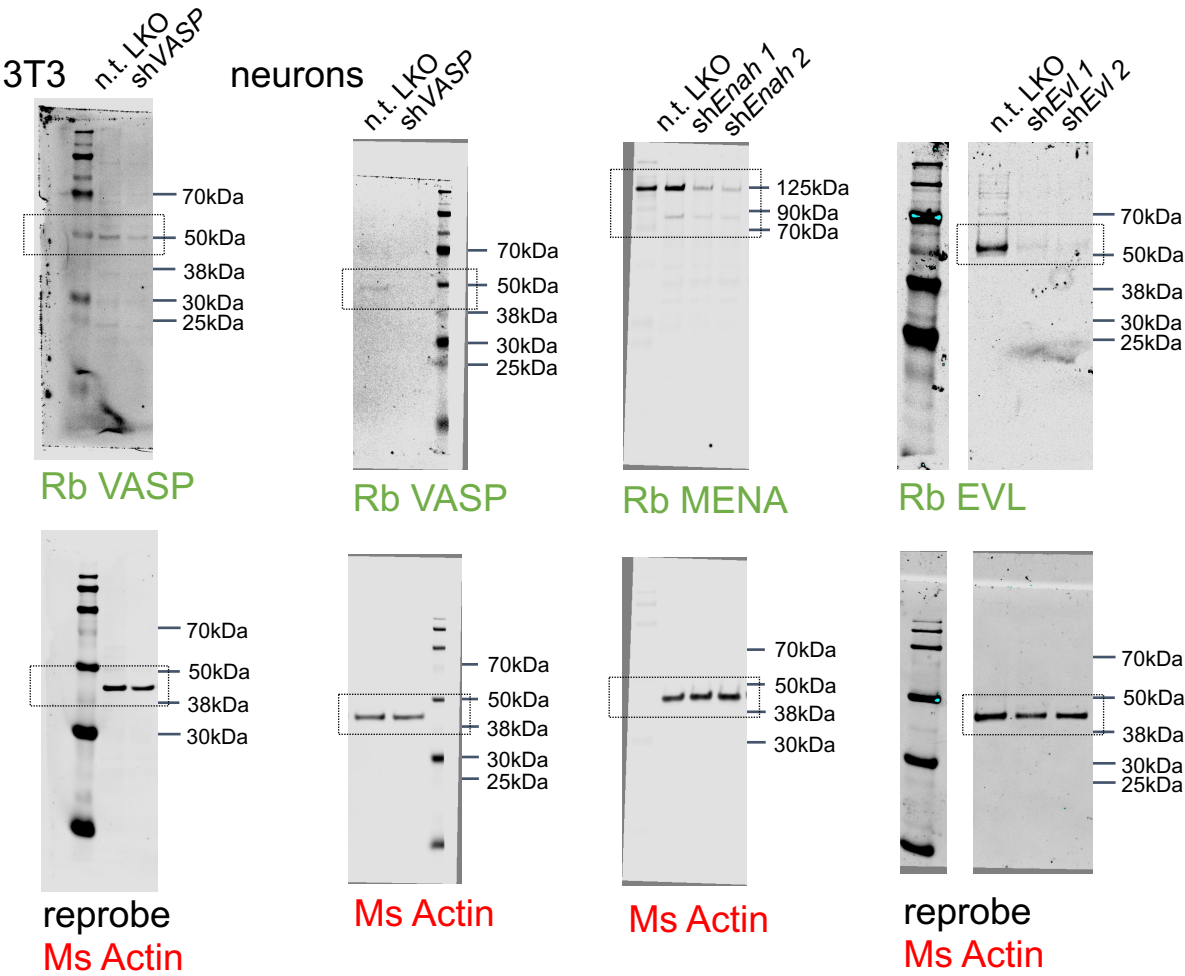

Supplement: SourceData FS2 — is the source file for Fig. S2. [file JCB_202106081_SourceDataFS2.pdf]

Fig S3A: MENA/EVL OE

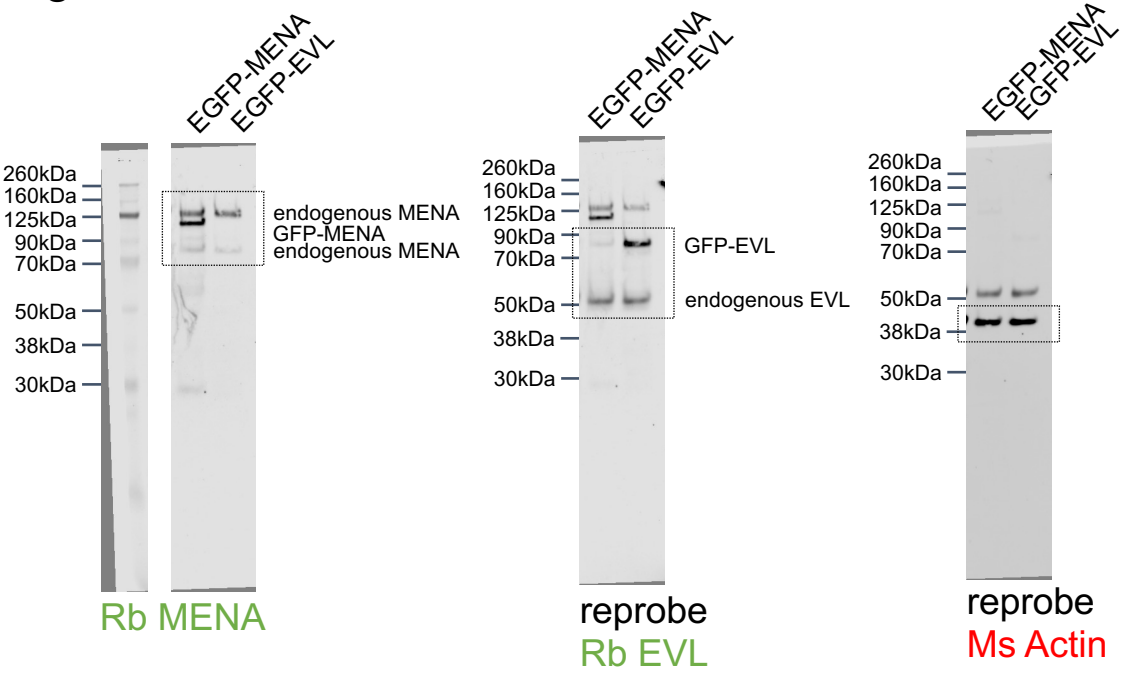

Supplement: SourceData FS3 — is the source file for Fig. S3. [file JCB_202106081_SourceDataFS3.pdf]

Fig S5F: WT vs EVL KO Expression

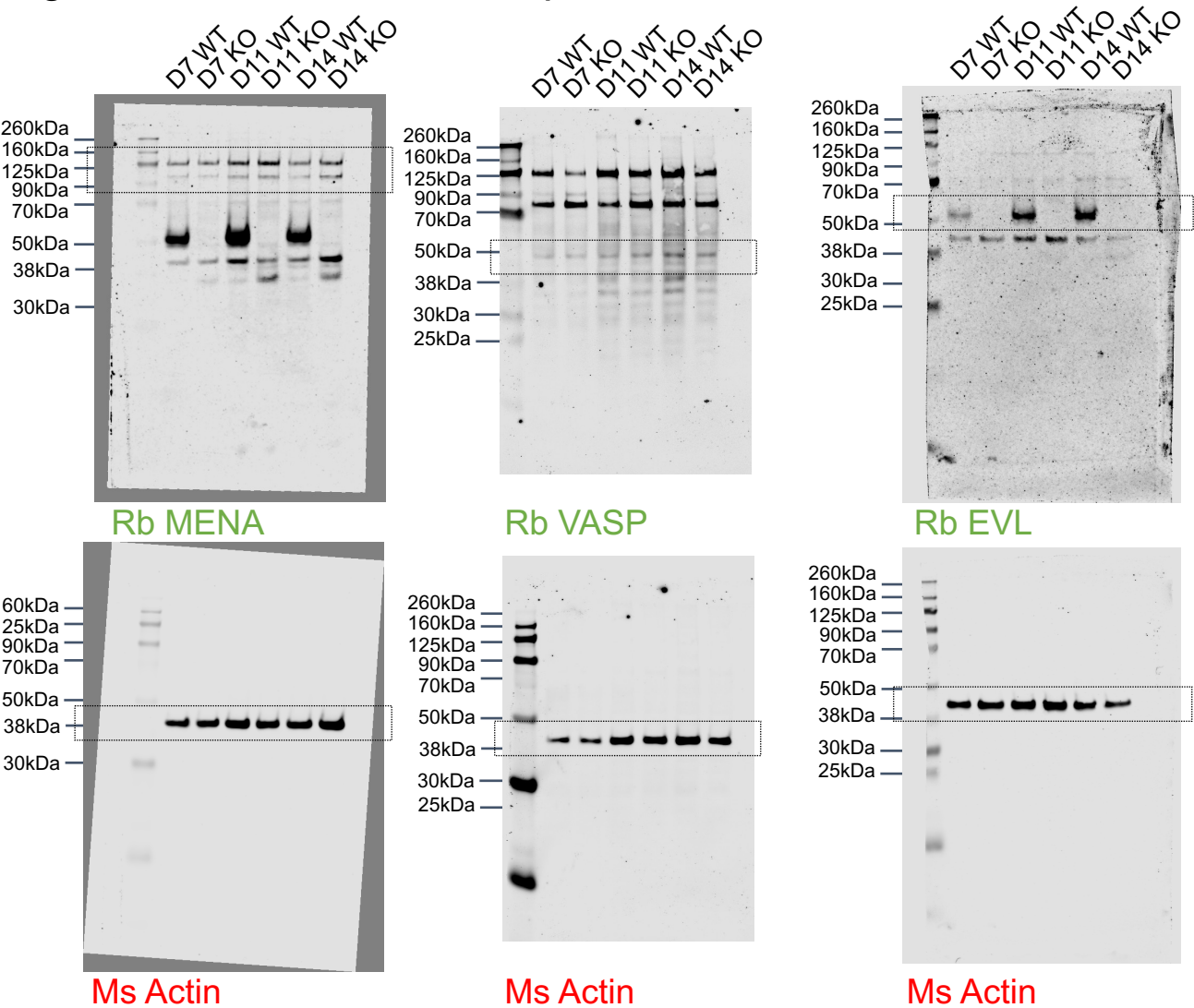

Supplement: SourceData FS5 — is the source file for Fig. S5. [file JCB_202106081_SourceDataFS5.pdf]

Fig S7B: Co-IPs of MIM and EVL

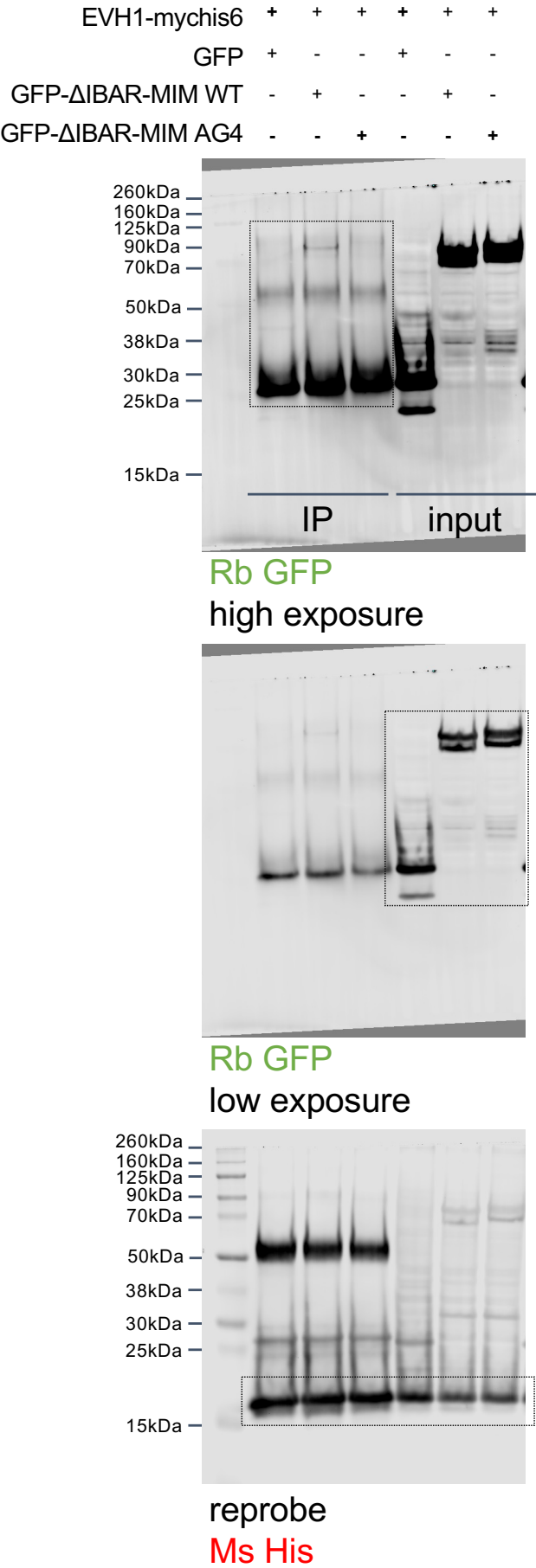

Fig S7C: MIM expression in cultured neurons

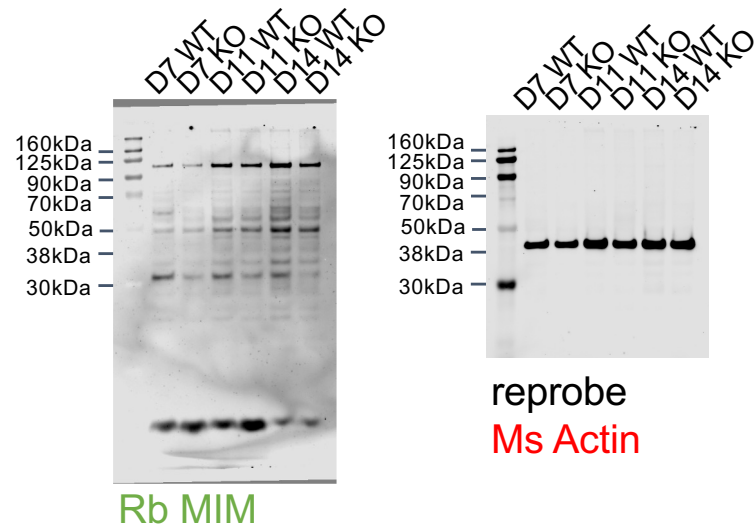

Fig S7D: MIM shRNA

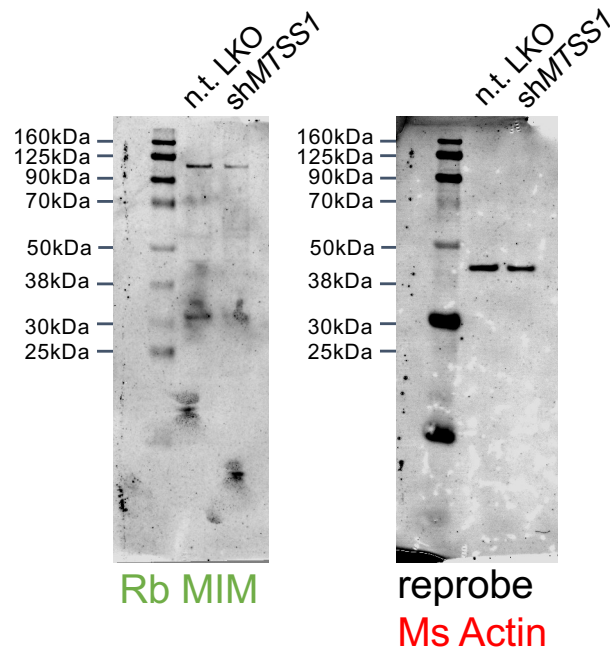

Supplement: SourceData FS7 — is the source file for Fig. S7. [file JCB_202106081_SourceDataFS7.pdf]
