# Supplementary material for: EVL and MIM/MTSS1 regulate actin cytoskeletal remodeling to promote dendritic filopodia in neurons
Source: J Cell Biol. 2023 Feb 24;222(5):e202106081. doi: 10.1083/jcb.202106081 (PMC9998662; doi:10.1083/jcb.202106081)

Fig 7AB: Co-IPs of MIM and EVL

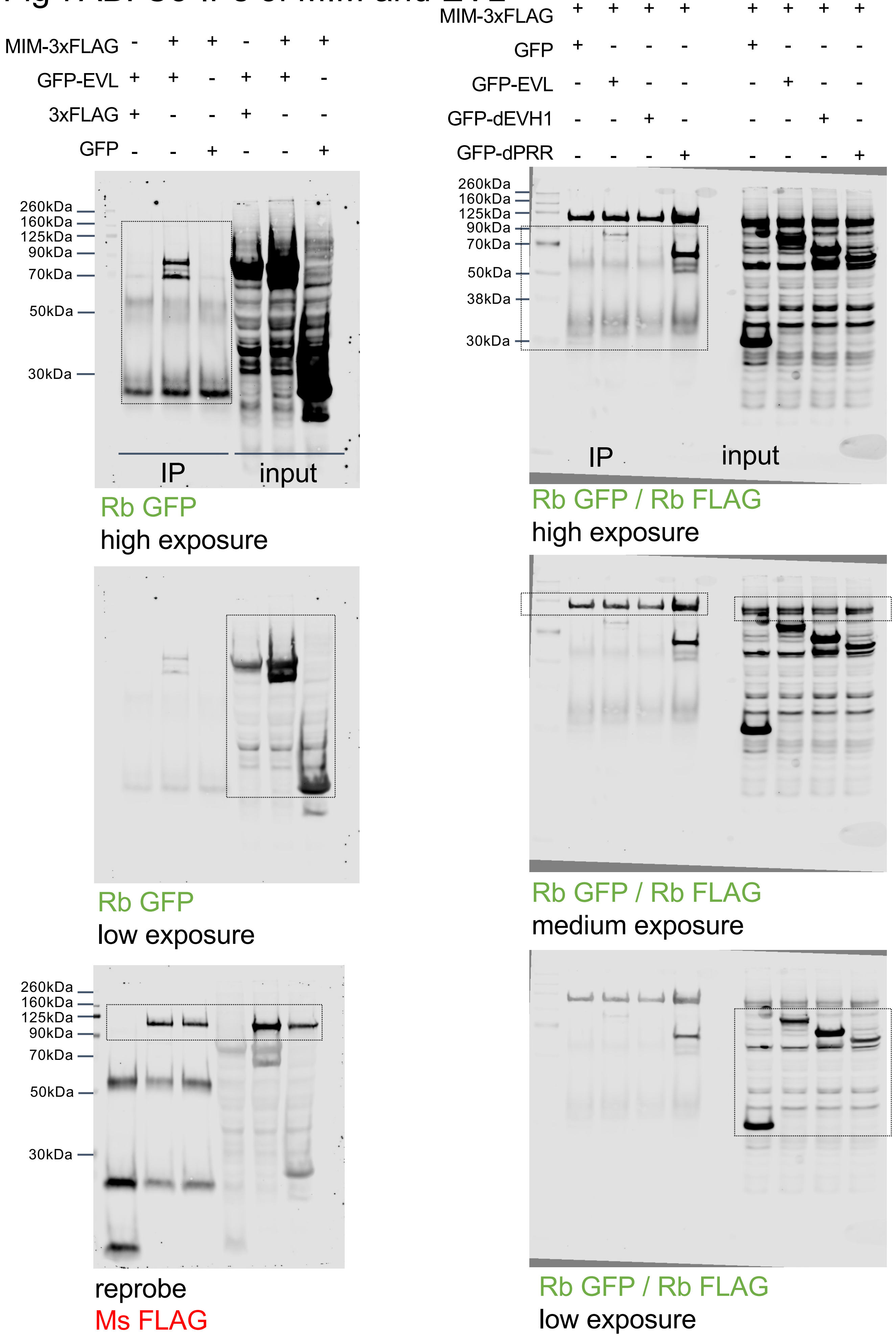

Supplement: SourceData F7 — is the source file for Fig. 7. [file JCB_202106081_SourceDataF7.pdf]
